# The Carboxyl-Terminal Amino Acids Render Pro-Human LC3B Migration Similar to Lipidated LC3B in SDS-PAGE

**DOI:** 10.1371/journal.pone.0074222

**Published:** 2013-09-10

**Authors:** Wei Wang, Zhixia Chen, Timothy R. Billiar, Michael T. Stang, Wentao Gao

**Affiliations:** 1 College of Animal Science and Veterinary Medicine, Jilin University, Jilin, China; 2 Department of Surgery, University of Pittsburgh School of Medicine, Pittsburgh, Pennsylvania, United States of America; Consejo Superior de Investigaciones Cientificas, Spain

## Abstract

LC3 is widely used marker for macroautophagy assays. After translation pro-LC3 is processed by Atg4 to expose C-terminal glycine residue for downstream conjugation reactions to accomplish the conversion of LC3-I to LC3-II. SDS-PAGE based Western blot (Wb) is generally utilized to quantify LC3-II levels where the LC3-I band migrates slower than LC3-II. We found that pro-human LC3B migrated at similar rate as LC3B-II in SDS-PAGE. The carboxyl-terminal five amino acids, particularly Lysine^122^ and Leucine^123^ of human LC3B play a major role in the faster migration of unprocessed LC3B, rendering it indistinguishable from LC3B-II in Wb assays. The unique faster migration of unprocessed LC3B than LC3B-I is also revealed in mouse LC3B, rat LC3B and rat LC3 but not in human LC3C. Our findings for the first time define pro-LC3 migration patterns for LC3 family member from human, mouse and rat species in SDS-PAGE. These findings provide a reference for pro-LC3 band patterns when Atg4 function is inhibited.

## Introduction

Macroautophagy (simply referred to as autophagy) is a highly conserved cellular degradation process. In contrast to the ubiquitin-proteasome degradation system, two ubiquitin-like conjugation systems are essential for autophagy: Atg12-Atg5 and Atg8/LC3- phosphatidylethanolamine (PE) [1], [2].

Atg8/LC3 lipidation involves a cysteine protease, Atg4, to process pro-LC3 exposing a C-terminal glycine residue to generate LC3-I. LC3-I is activated by Atg7 as a thioester bond is formed between a catalytic cysteine residue of Atg7 and the C-terminal glycine of LC3-I. LC3-Atg7 intermediate afterward exchanges with Atg3, an E2-like enzyme, to form a second thioester intermediate, LC3-Atg3 [3]. Finally, the lipidation process is completed by the putative E3-like enzyme Atg16L1•Atg5-Atg12 complex to form LC3-II, as LC3-Atg3 is exchanged with PE [4].

Mammalian cells have several LC3 isoforms (MAP1LC3A, B, B2 and C, simply referred as to LC3A, B, B2 and C) and paralogues (GABARAP, GABARAPL1, and GATE-16) [5]. Four Atg4 homologues (Atg4A, Atg4B, Atg4C, and Atg4D) are identified in mammalian cells. Atg4B cleaves LC3, GATE-16, and GABARAP, with the highest affinity for LC3 [5]. Atg4A is preferential for GABARAP and GATE-16 [6].

Although mammalian cells contain several variants of LC3 and paralogues, LC3B is the most widely used marker in autophagic assays as LC3B is expressed in nearly all tissues [7]. If not specifically described, most of LC3 Wb assays are based up LC3B in the publications. Indeed, most LC3 antibodies commercially available for LC3-I/LC3-II conversion assays are generated based on LC3B. By Wb assay, there are two distinguishable bands recognized as LC3-I (slower moving band) and LC3-II (faster moving band) [8]. The ratio between LC3-II and LC3-I or an appropriate loading control is generally regarded as an indicator and measure for the overall cellular process of autophagy.

Replacement of the Glycine120 residue within LC3B (G120A) abolishes LC3B cleavage by Atg4B [9]. Of interest, we have found that the G120A mutation of human LC3B actually migrated to a similar site as LC3B-II by SDS-PAGE. Initially, we considered that LC3B^G120A^ might be cleaved by an unknown enzyme *in vivo* explaining its altered migration behavior; however, after sequencing purified LC3B^G120A^ by MALDI ISD, we found that it proved to be the full-length protein. We question whether unprocessed human LC3B migrates at a similar rate to that of LC3B^G120A^ in SDS- polyacrylamide gel. We found that the last five amino acids in human LC3B alter unprocessed LC3B migration behavior in SDS-PAGE. The consequence of this unique property of human LC3B leads to pro-LC3B indistinguishable from LC3B-II in Wb assay. The unique character of C-terminal amino acids after the Glycine conjugation site renders pro-LC3B faster migration than LC3B-I was furthermore revealed in mouse LC3B, rat LC3B, and rat LC3.

## Materials and Methods

### Reagent and Antibodies

Cell culture reagents were purchased from LONZA (Walkersville, MD). The following antibodies were used: Rabbit anti- LC3B antibody (Cell Signaling, raised by a synthetic peptide from N-terminal 2-25aa); Rabbit anti- LC3A antibody (abcam, raised by a synthetic peptide from N-terminal 2-15aa); Rabbit anti- LC3C antibody (Abnova, raised by a synthetic peptide from N-terminal 2-30aa); mouse anti-GFP antibody (Santa Cruz biotech); mouse anti- FLAG M2 and β-actin antibodies (Sigma-Aldrich); and Goat anti-rabbit IgG (HRP-conjugated) and Goat anti-mouse IgG (HRP-conjugated) (Jackson ImmunoResearch). Recombinant His_6_-Atg4B was from R&D system (E-400). Atg4B siRNA and Control siRNA are from Cell Signaling. All other reagents were purchased from Sigma-Aldrich.

### Cell Culture

HEK293 (ATCC), A549 cells (ATCC), wild-type MEF cell (MEFwt) and Atg7 knockout MEF cell (MEFatg7KO) were provided by Dr. Masaaki Komatsu, and have been described [10]. Cells were grown in DMEM supplemented with 10% fetal bovine serum, 2 mM L-glutamine, and 100 U/ml penicillin/streptomycin in a 5% CO2 incubator at 37°C.

### LC3 Gene Expression Vectors

Human MAP1LC3B Open Reading Frame (ORF) with N-terminal Myc-tag, 3xFlag tag or GFP tag was cloned into an expression vector by PCR using cDNA from HEK293 cell. Point mutations were generated by site-directed mutagenesis (Invitrogen) on these vectors as described in the results section. Truncates of LC3B were generated based on 3xFlag tagged LC3B vector by introducing a stop codon after indicated amino acid at C-terminal as described in the results section by site-directed mutagenesis. 3xFlag tagged LC3B and its mutant ORFs were sub-cloned into pTriEx-1.1 (Novagen) to analyze their expression patterns in mammalian and *E.coli* cells, respectively. None tagged ORFs for Human LC3B and LC3C, and mouse LC3B and LC3A, and Rat LC3B and Rat LC3 and the correspondent mutants were inserted into pTriEx1.1. All inserts were validated by DNA sequencing.

Adenoviral vectors Ad-GFP-LC3B, Ad-GFP-LC3B^G120A^, Ad-GFP-LC3B^G120E^ and Ad-GFP-LC3B^G120D^ were generated using pAdlox system and purified as described [11].

### Recombinant Protein Purification and Sequencing

3xFlag-LC3B^G120A^ expression plasmid was transfected into HEK293 cells by lipofectamine 2000 (Invitrogen). 3xFlag-LC3B^G120A^ recombinant proteins were purified 48 hours later post transfection using total cell lysate by Flag antibody (M2) based affinity column, according to the suggested protocol. Eluted 3xFlag-LC3B^G120A^ proteins were analyzed by 12% of SDS-PAGE and stained with PageBlue Protein Staining Solution (Thermo Scientific). The N-terminal and C-terminal sequences of the purified proteins were determined by top-down protein sequencing by MALDI ISD mass spectrometry of intact proteins (Laboratory for Proteomic Mass Spectrometry, University of Massachusetts Medical School). Analyses were performed on a Shimadzu Biotech Axima TOF^2^ (Shimadzu Instruments) matrix-assisted-laser desorption/ionization Time-of-Flight (MALDI-TOF) mass spectrometer. Proteins were analyzed in positive ion Linear mode. For intact protein mass measurement the instrument was set with a mass range extending to 50 kd using a pulsed extraction setting of 16952. Apomyoglobin MH+ of 16952 and MH2+ of 8476 were used as the standard to calibrate the instrument. A 0.5 µl aliquot was applied to the MALDI target followed by the addition of 0.5 µl of Sinapinic acid as the desorption matrix (10 mg/ml in 0.1%TFA: Acetonitrile 50∶50) followed by air drying prior to insertion into the vacuum source. For In-Source-Decay (ISD) sequencing the instrument was set to Linear positive ion mode with a pulsed extraction setting of 2500. Spectra were acquired using 1, 5-Diaminonaphthalene (10 mg/ml in 0.1%TFA: Acetonitrile 50∶50) as the matrix. Samples were applied in the same way as the Sinapinic acid matrix. For database searching selected ISD fragments (* in the spectra) were used as virtual precursors and submitted to the Mascot search engine MS/MS ion search program (Matrix Science, Ltd. Version 2.3) through the Shimadzu Biotech Launchpad software. For Z ion series a Mascot modification of “amino loss N-term” was created to accommodate the structure of the Z ion when selecting fragment precursors.

### Expression of Flag Tagged or None Tagged LC3B and its Mutants in HEK293 and *E.coli*

pTriEx 1.1 expression vector based Flag tagged LC3B and none tagged LC3B and its mutants were expressed in HEK293 for 24 hours or in Tuner™(DE3)pLacI *E. coli* (Clontech) by induction of expression with 1 mM of IPTG for four hours. Cell lysates were separated by15% of SDS-polyacrylamide gels and analyzed by immunoblotting with Flag or LC3B antibodies.

### Atg4B Cleavage Assay

Flag tagged human LC3B, LC3B and its mutants in pTriEX1.1 expression vectors were transformed into Tuner™(DE3) pLacI cells. Colonies were picked and incubated in 4 ml of LB media with 1% of glucose overnight in a shaker at 37°C. 500 µl of overnight bacteria culture was then added into a new tube with 3 ml fresh LB media and incubated in shaker at 37°C for three hours. 1 mM of IPTG was added to induce LC3B expression for three hours. Bacteria were then collected and washed once with ice cold phosphate-buffered saline. Pellets were resuspended in RIPA buffer (50 mM Tris HCl pH8, 150 mM NaCl and 1% NP-40) and sonicated. Cell lysate was centrifuged at 10,000g for 15 min at 4°C. 10 µg of supernatant was used for Atg4B cleavage assay in 50 µl digestion buffer (25 mM Tris-HCl, pH7.4, 50 mM KCl) with 0.2 µg of Atg4B at 37°C. Digestion mixture was collected at different time point and analyzed by immunoblotting using15% of SDS-polyacrylamide gels.

### Fluorescence Microscopy

A549, MEFwt or MEFatg7KO cells were infected with adenoviral vectors expressing GFP-LC3B, GFP-LC3B^G120A^, GFP-LC3B^G120E^, or GFP-LC3B^G120D^ at 5 MOI. Twenty four hours post infection cells were treated with 100 µM of Chloroquine for two hours to induce GFP-LC3B punctation. Images were then recorded using EVOSfl fluorescence microscopy.

### Western Blotting

Cells were transfected or infected with expression plasmid vectors or viral vectors. Cells were rinsed with ice-cold phosphate-buffered saline twenty-four hours later, scraped, and collected by centrifugation at 4°C and lysed in RIPA lysis buffer (Cell Signaling) with protease inhibitor cocktails (Sigma Aldrich). Cell lysates were centrifuged at 15,000 *g* for 15 min at 4°C, and supernatants were collected. 15 µg of cell lysates were separated by 15% of SDS-polyacrylamide gels and transferred to reinforced NC membrane (Whatman GmbH). Blots were probed with primary antibodies and detected with horseradish peroxidase (HRP)-conjugated anti-mouse or anti-rabbit IgG (Jackson ImmunoResearch). Bands were visualized using SuperSignal West Pico Substrate (Thermo Scientific).

## Results

### Human LC3B^G120A^ Migrates at a Similar Rate as LC3B-II Specie in SDS-PAGE

**Figure 1:**
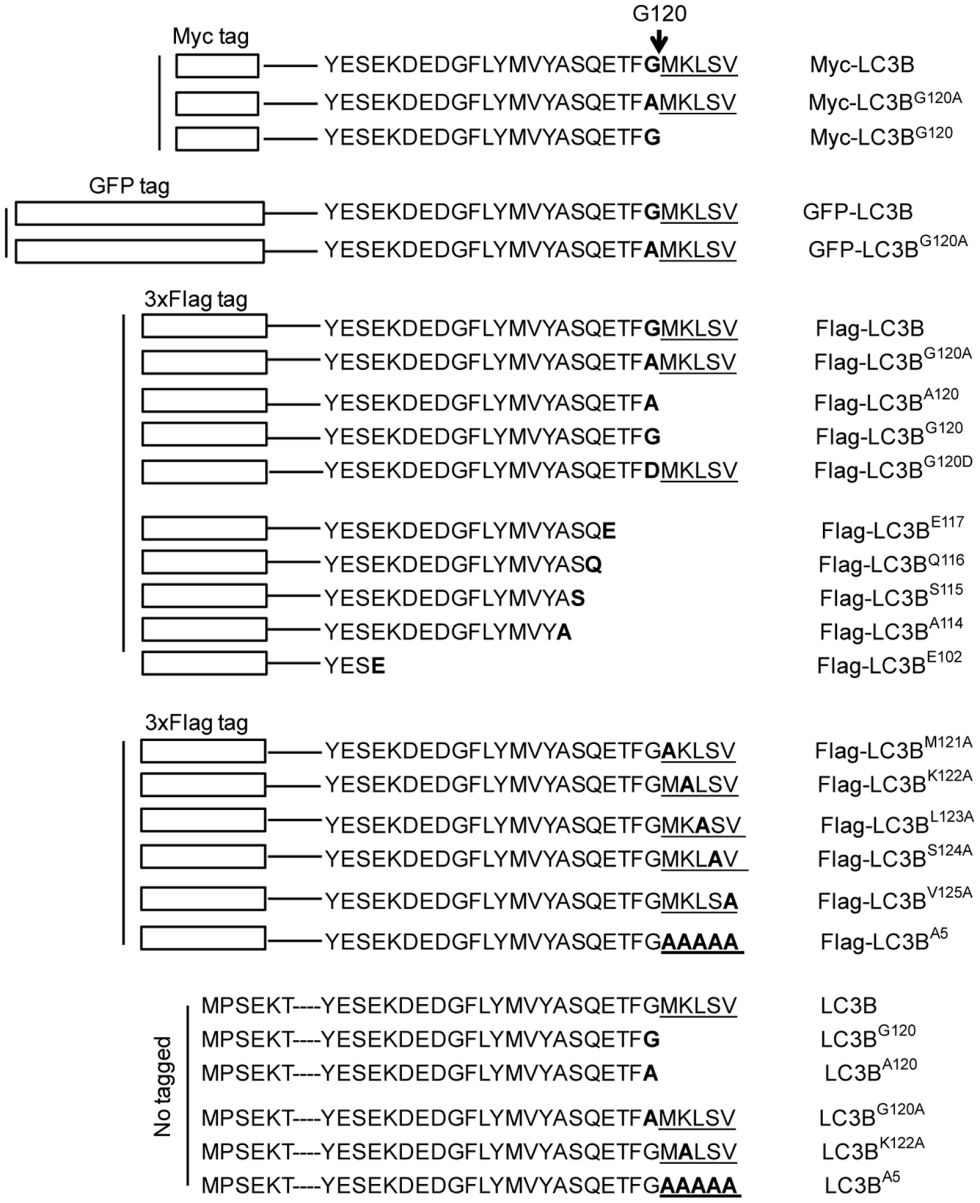
Schematic diagram of expression plasmid constructs. Myc, GFP, or 3xFlag tag is fused to human MAP1LC3B ORF at its N- terminus. The box bars on the left represent the N-terminal tags. The dashed lines represent MAP1LC3B with 27 amino acids sequence shown at the carboxyl terminus. The arrow points to Glycine 120 conjugation site. The last five amino acids at the C-terminal are underlined. Mutant amino acid or last amino acid of LC3B is bolded. Human LC3C, Rat LC3, Rat LC3B, Mouse LC3A, Mouse LC3B and the related mutants are described in Result section.

**Figure 2:**
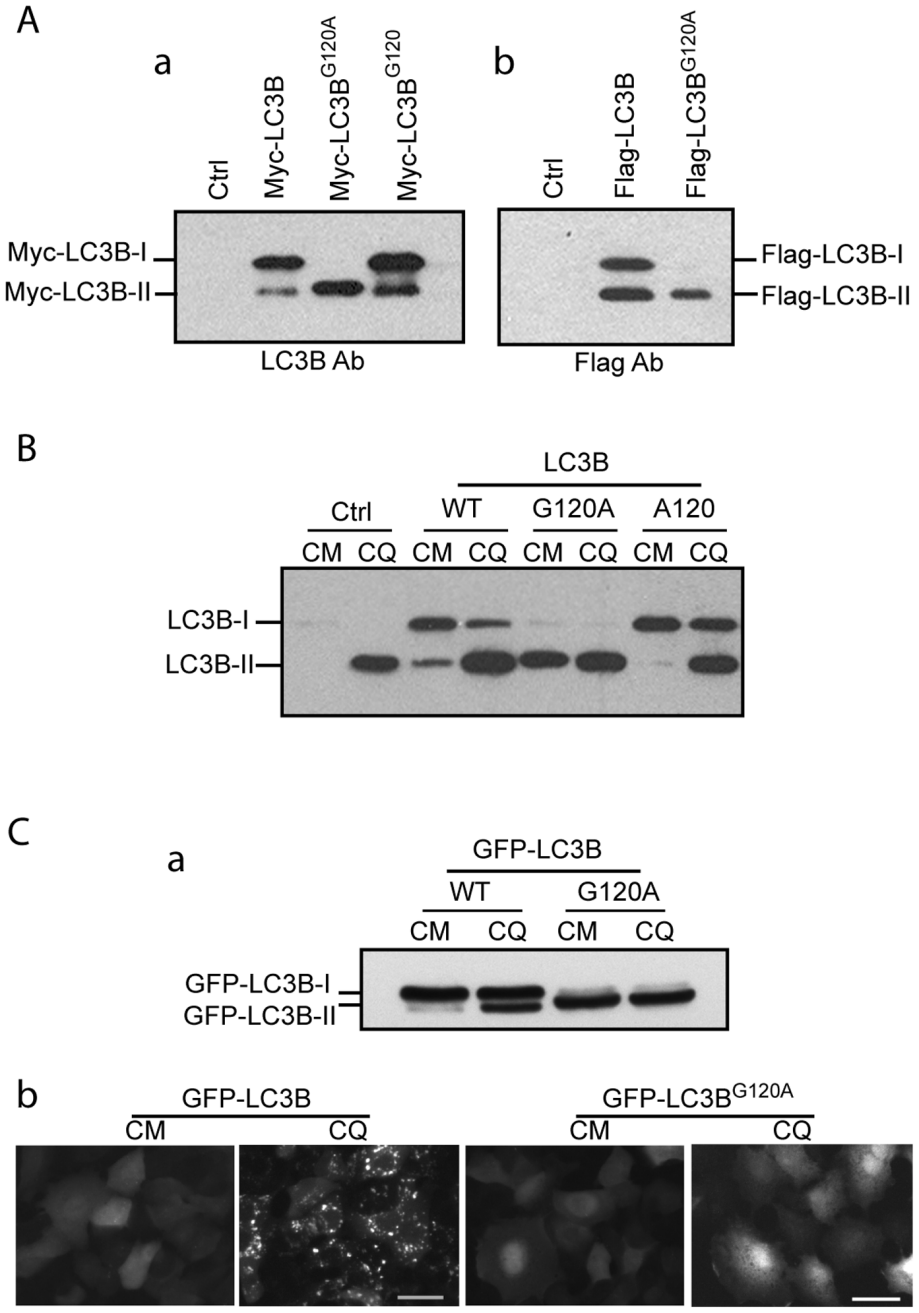
G120A mutant of human LC3B has similar mobility to LC3B-II in SDS-polyacrylamide gel. ***Aa***, plasmids expressing Myc-LC3B, myc-LC3B^G120A^ or Myc-LC3B^G120^ was expressed in HEK293 cells for 24 hours. Cells were harvested and lysed by sonication, and 10 µg of total protein of each lysate were separated by 15% of SDS-polyacrylamide gel, followed by immunoblotting with anti-LC3B antibody. ***Ab,*** plasmids expressing Flag-LC3B or Flag-LC3B^G120A^ was expressed in HEK293 cells for 24 hours. Cells were lysed followed by immnuoblotting using Flag-antibody. ***B,*** plasmids expressing LC3B, LC3B^G120A^ or LC3B^A120^ was expressed in HEK293 cells for 24 hours. Cells were treated with 50 µM of CQ for 2 hours before collection. Transient expressed proteins were analyzed by immunoblotting using LC3B antibody.***Ca,*** plasmids expressing GFP-LC3B or GFP-LC3^G120A^ was expressed in HEK293 cells for 24hours. Cells were then treated with 50 µM of Chloroquine (CQ) for 2 hours. Wb assays were performed using anti-GFP antibody. ***Cb,*** A549 cells infected with Adenoviral vectors expressing GFP-LC3B or GFP-LC3B^G120A^ at 5 MOI for 24 hours. Cells were then treated with 50 µM of CQ for two hours. GFP-LC3B punctuation was recorded by fluorescence microscopy. CM, complete media, CQ, complete media plus CQ. Scale bar: 25 micron.

Expression vectors of human LC3B used in this study are listed in Figure 1. Other expression vectors used in this study are described in Materials and Methods section and Results section, respectively. Human LC3B must be cleaved at the Gycine 120 position by Atg4B prior to the sequential formation of conjugates with Atg7 and Atg3 in the process of LC3B-PE conjugation. N-terminal Myc-tagged human LC3B demonstrated two bands in SDS-PAGE based Wb assay (Fig. 2Aa, lane 1) corresponding to Myc-LC3B-I (cleaved form) and Myc-LC3B-II (lipidated form). A truncated Myc-LC3B^G120^ also demonstrated Myc-LC3B-II species (Fig. 2Aa, lane3) indicating both wild-type and G^120^ mutant of Myc tagged LC3B undergo the expected steps for lipidation with PE. We unexpectedly observed the Myc-LC3B^G120A^ mutant migrated at a similar rate as Myc-LC3B-II (Fig. 2Aa, lane 2). This was confirmed utilizing alternate 3xFlag tagged human LC3B and LC3B^G120A^ vectors (Flag-LC3B and Flag-LC3B^G120A^, respectively). Again, Flag-LC3B^G120A^ migrated at a similar rate as Flag-LC3B-II (Fig. 2Ab).

These data are furthermore verified in non-tagged human LC3B expression vectors. Transient expression of un-tagged wild-type LC3B demonstrated the expected LC3B-I (major band) and LC3B-II (minor band) species in untreated HEK293 cells (Fig. 2 B, Lane 3) and increased LCB-II band intensity following treatment with chloroquine (CQ) (lane 4). In contrast, the G120A mutant of LC3B demonstrated a major band at a similar migration point to LC3B-II (Fig. 2B, Lanes 5 and 6). As the migration patterns of LC3B-II and LC3B^G120A^ bands are relatively indistinguishable, the increased band intensity observed in LC3B^G120A^ CQ treatment is likely due to the endogenous increase in LC3B-II as demonstrated in the untransfected control (Figure 2B, lane 2).

In consideration that LC3B^G120A^ has the same number of amino acids as the uncleaved LC3B, we hypothesized that LC3B^G120A^ could be cleaved or alternatively processed *in vivo* by an unspecified enzyme causing its faster migration. The truncated LC3B^A120^ represents a hypothetical G120A mutant “cleaved” at the A120 position similar to wild type LC3B cleavage at glycine 120 and serves as a control. Expression of LC3B^A120^ demonstrated band signal at a position similar to that of LC3B-I (Fig. 2B, Lane 7). Again, the increased signal at the point of LC3B-II migration observed in LC3B^A120^ CQ treatment is likely the result of endogenous increase in LC3B-II.

To determine whether LC3B^G120A^ can be lipidated and target autophagosome membrane, GFP tagged LC3B expression vectors were constructed. GFP-LC3B responded to treatment with CQ by demonstrating GFP-LC3B-II accumulation (Fig. 2Ca, lane 2). In contrast, the GFP-LC3B^G120A^ mutant demonstrated a similar SDS-PAGE migration rate as GFP-LC3B-II (Fig. 2Ca, lane3). CQ treatment had no effect on GFP-LC3B^G120A^ (Fig. 2 Ca, lane 4). As shown in Figure 2 Cb, CQ induced multiple punctae of GFP-LC3B while GFP-LC3B^G120A^ did not respond to CQ treatment. This finding indicates that GFP tagged wild-type LC3B passes normally through the lipidation process; however, the G120A mutant form is not associated with a lipidation process marked by the absence of puncta accumulation with CQ treatment.

Altogether, these data demonstrate that the G120A mutant of human LC3B migrates at a similar rate as LC3B-II in SDS-PAGE and this phenomenon is independent of N-terminal tags. Further, the faster migration of the G120A mutant does not likely represent an unexpected lipidation process.

### Analysis of Alternative Point Mutation at G^120^ Site and LC3B Truncates in SDS-PAGE

**Figure 3:**
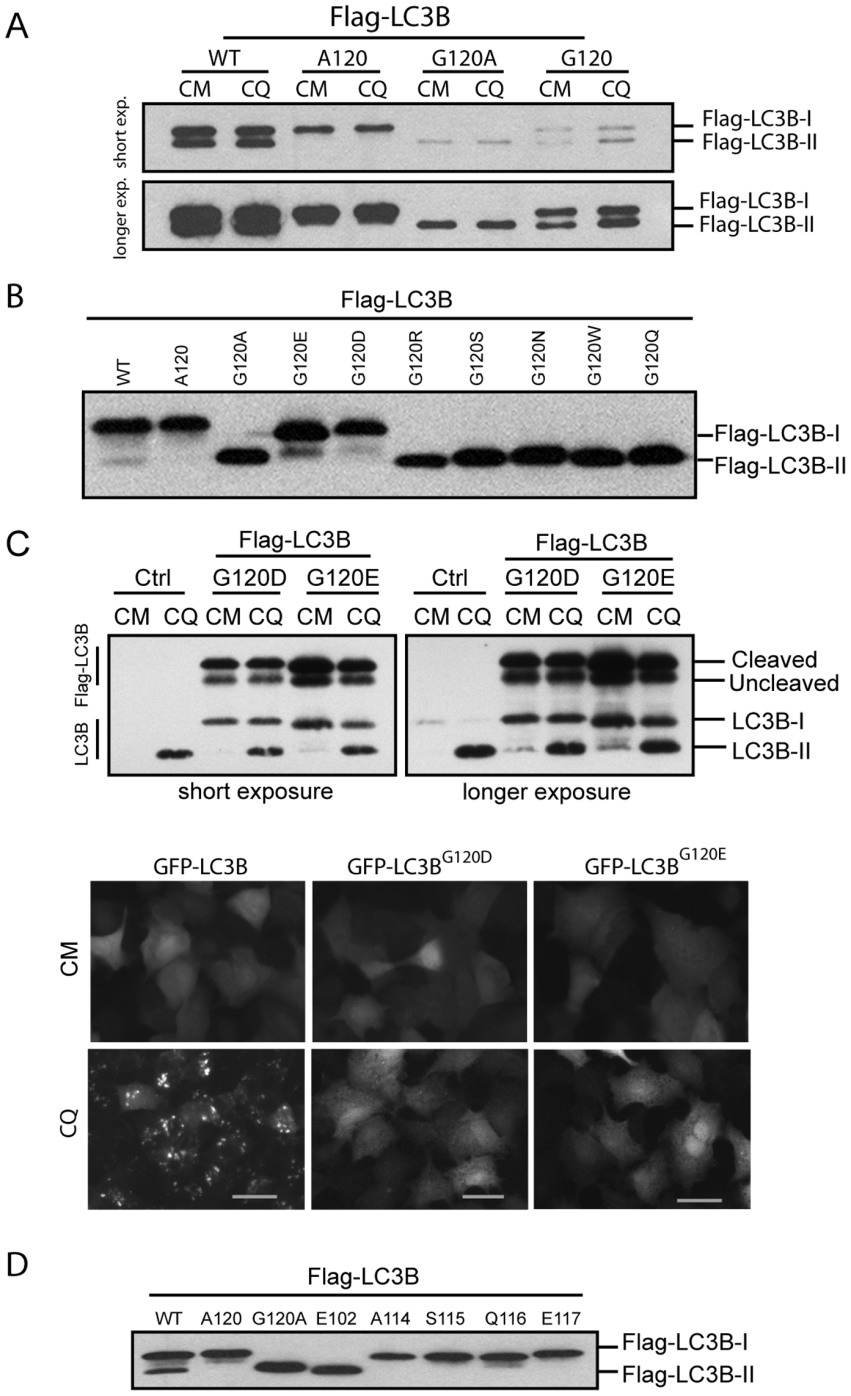
Analysis of different group amino acid replacement of Glycine 120 of human LC3B and LC3B truncates effect on their mobility in SDS-polyacrylamide gel. ***A,*** plasmids expressing Flag-LC3B, Flag-LC3B^G120A^, Flag-LC3B^G120^ or Flag-LC3^A120^ was expressed in HEK293 cells for 24 hours. Cells were treated with 50 µM of CQ for two hours and harvested and lysed. Total cell lysates (10 µg) was used for immunoblotting by Flag antibody. ***B,*** G120 point mutant plasmids were expressed in HEK293 cells for 24 hours. Immunoblotting was conducted to compare the band’s mobility with Flag-LC3B, Flag-LC3B^G120A^ or Flag-LC3B^G120A^ as indicated. ***C,*** Flag-LC3B^G120D^ and Flag-LC3B^G120E^ were expressed in HEK293 cells for 24 hours. Cells were then treated with 50 µM of CQ for 2 hours. Expression patters of Flag-LC3B^G120D^ and Flag-LC3B^G120E^ were compared with endogenous LC3B by immunoblotting with LC3B antibody (upper panels). Adenoviral vector expressing GFP-LC3B, GFP-LC3B^G120D^ or GFP-LC3B^G120E^ was used to infect A549 cells for 24 hours. Cells were then treated with 50 µM of CQ for 2 hours. Images were recorded using fluorescence microscopy. Scale bar: 25 micron. ***D,*** Truncates of LC3B (see Fig. 1) were expressed in HEK293 for 24hours. Cells were treated with 50 µM of CQ for two hours before harvesting. Total cell lysates were used for Wb assay to compare the mobility with Flag-LC3B-I and Flag-LC3B-II. Flag antibody was used to detect the recombinant proteins.

The unexpected human LC3B^G120A^ SDS-PAGE migration behavior prompted us to characterize LC3B in Wb assays. A series Flag tagged human LC3B wild-type and mutant expressing vectors were generated. As expected, Flag-LC3B showed clear Flag-LC3B-I and Flag-LC3B-II bands (Fig. 3A, WT). A Flag-LC3B^G120^ vector was generated by removing the C-terminal five amino acids of human LC3B. This recombinant protein bypasses Atg4B cleavage and undergoes expected downstream reactions with Atg7 and Atg3 as LC3B-I. Flag-LC3B^G120^ demonstrated two bands by Wb immunoassay (Fig. 3A, G120). The upper band corresponds to Flag-LC3B^G120^ (size equivalent to Flag-LC3B-I). The lower band corresponds to Flag-LC3B-II. CQ treatment increased Flag-LC3B-II level. This result demonstrates that Flag-LC3B^G120^ is lipidated in a normal fashion. A Flag-LC3B^A120^ vector was constructed by removing the last five amino acids of human LC3B and replacing Glycine 120 with Alanine. Thus, this recombinant protein would be anticipated to not pass through the lipidation process. Flag-LC3B^A120^ is equal in size to Flag-LC3B-I or Flag-LC3B^G120^ and migrates similarly by Wb (Fig. 3A, A120). CQ treatment did not induce Flag-LC3B-II accumulation with the Flag-LC3B^A120^ recombinant protein. Again, Flag-LC3B^G120A^ (Fig. 3A, G120A) migrated to a similar position as Flag-LC3B-II. As CQ had no effect on band intensity of Flag-LC3B^G120A^ this indicates Flag-LC3B^G120A^ does not pass through the lipidation process. Overall, these data confirmed the finding already shown in Figure 2.

Utilizing a set of Flag tagged LC3B vectors, we investigated the effect of amino acid replacement at the G120 position on LC3B migration by SDS-PAGE. A series of amino acid replacements at the Glycine 120 position were constructed. Flag-LC3B^G120Q^, -LC3B^G120N^, -LC3B^G120S^, -LC3B^G120W^, and -LC3B^G120R^ demonstrated migration rates similar to Flag-LC3B^G120A^ (Fig. 3B). However, Flag-LC3B^G120E^ and -LC3B^G120D^ appeared to be cleaved and resulted in a major band similar to Flag-LC3B-I with the addition of a faster migration band between that of Flag-LC3B-I and Flag-LC3B-II. This indicates that LC3B with acidic group amino acids (D or E) at the G120 site were also likely cleaved.

The characteristics of the faster moving species in Flag-LC3B^G120D^ and Flag-LC3B^G120E^ were analyzed. Should cleaved Flag-LC3B^G120D^ or –LC3B^G120E^ pass through a lipidation process, one should observe the accumulation of the faster moving species with CQ treatment. The faster migration species should target autophagosome membrane and form puncta under stimulation. As shown in Fig. 3C, there was no discernable increase in the faster migration species with either Flag-LC3B^G120D^ or –LC3B^G120E^ under CQ treatment; whereas, endogenous LC3B-II clearly accumulated, indicating that Flag-LC3B^G120D^ or –LC3B^G120E^ do not pass through downstream lipidation reactions even though it can be cleaved. This conclusion was further supported by GFP tagged LC3B^G120D^ or LC3B^G120E^ showing absence of puncta formation with CQ treatment compared to puncta formation in the GFP tagged wild-type LC3B control (Fig. 3 C, lower panels).

A systematic series of Flag-LC3B truncates were constructed by removing C-terminal amino acids to experimentally predict a potential “cleavage site” for Flag-LC3B^G120A^ should its faster migration actually represent an aberrant cleavage processing event. The LC3B truncates followed expected principles of denatured protein electrophoresis by SDS-PAGE and migration was mitigated by their respective sizes (Fig. 3D), i.e., the longer the truncated protein, the slower the migration. Flag-LC3B^E102^ with 23 amino acids removed from C-terminal of LC3B demonstrated a similar migration rate to that of Flag-LC3B^G120A^.

Altogether, these results demonstrate that in general alteration of the amino acid residue at G120 changed the migration of LC3B in SDS-PAGE and should this alteration induce an unexpected alternate protein cleavage it approximates around the site of E102 of human LC3B.

### Sequencing of Flag-LC3B^G120A^ Reveals it to be a Full Length Protein without Cleavage

**Figure 4:**
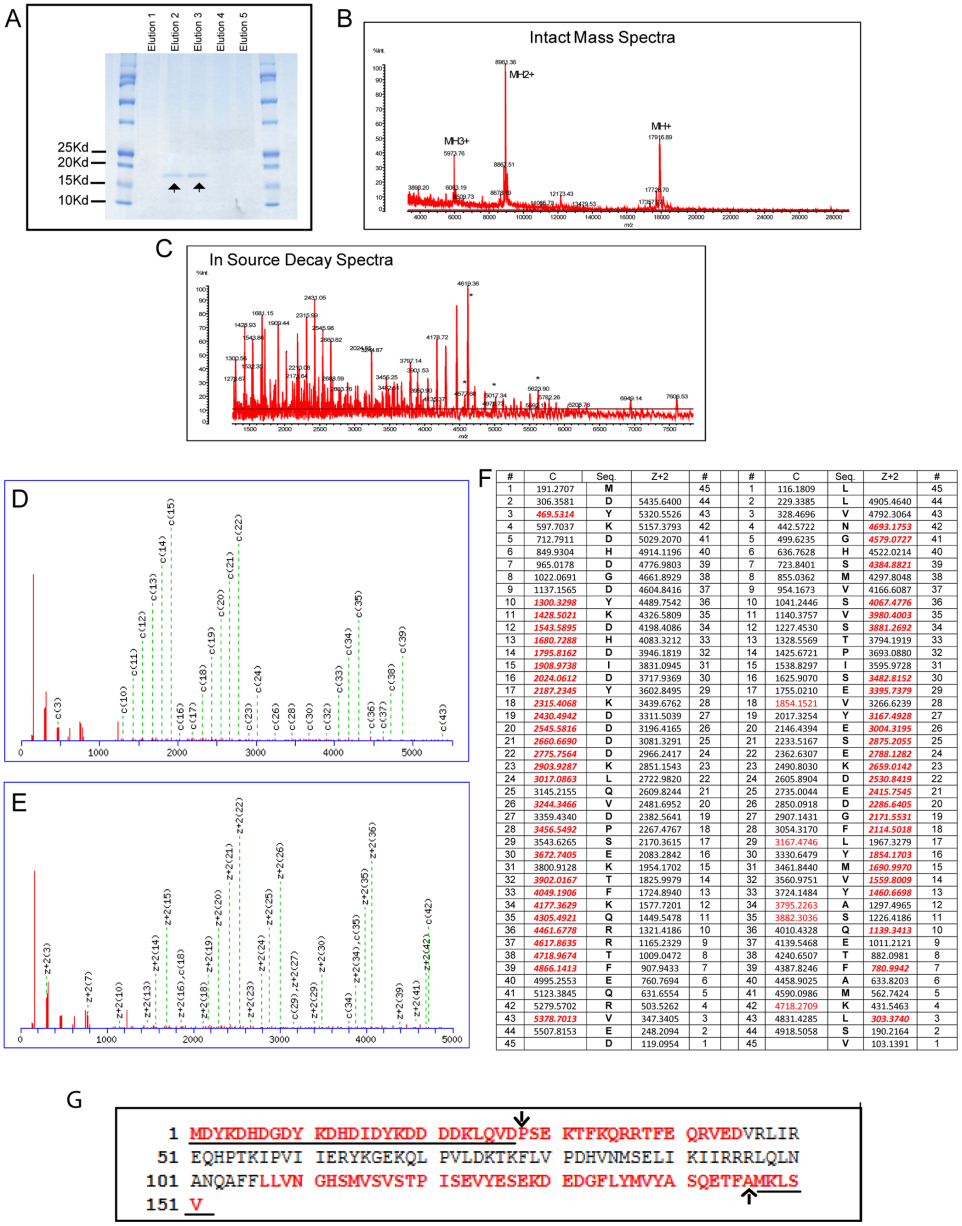
Sequence analysis of Flag-LC3B^G120A^ by MALDI ISD. Flag-LC3B^G120A^ expression vector was transfected into HEK293 cell for 48 hours. Cells were harvested and lysed with protease inhibitor cocktail. The lysates were centrifuged at 10,000 g for 30 min. Supernatants were then used for affinity purification of Flag-LC3B^G120A^ by Flag antibody conjugated beads in column according to the proposed protocol. ***A,*** 20 µl of elution (from total 1 ml) from Flag antibody based affinity column was loaded onto 12.5% of SDS-polyacrylamide gel. Gel was stained with PageBlue Protein Staining Solution. Elution number 2 and 3 were combined and concentrated. Purified Flag-LC3B^G120A^ was used to run MALDI ISD. Intact mass spectra are shown in ***B*** and in-source decay spectra are shown in ***C***. For database searching selected ISD fragments (* in the spectra) were used as virtual precursors and submitted to the Mascot search engine MS/MS ion search program (Matrix Science, Ltd. Version 2.3) through the Shimadzu Biotech Launchpad software. ***D,*** peptide view of Mascot Search results MS/MS Fragmentation of N-terminal: MDYKDHDGDYKDHDIDYKDDDDKLQVDPSEKTFKQRRTFEQRVED (Found in IPI00953817; Data file: Automatically uploaded data; Average mass of neutral peptide Mr(calc): 5622.8801; Ions Score: 168 Expect: 2.1e-012; Matches : 28/88 fragment ions using 47 most intense peaks.). ***E,*** peptide view of Mascot Search results: MS/MS Fragmentation of C-terminal: LLVNGHSMVSVSTPISEVYESEKDEDGFLYMVYASQETFAMKLSV(Found in IPI00953817; Data file Automatically uploaded data; Average mass of neutral peptide Mr(calc): 5017.6143; Ions Score: 34 Expect: 53;Matches : 30/88 fragment ions using 134 most intense peaks.). ***F,*** Theoretical fragment ions for the peptide sequence. The matched fragment ions are highlighted in red typed. ***G,*** Protein view: Match to: IPI00953817 Score: 297. Found in search of Automatically uploaded data. Nominal mass (M_r_): 17870; Calculated pI value: 5.38. NCBI BLAST search of IPI00953817 against nr Unformatted sequence string for pasting into other applications. Sequence Coverage: 59%. Matched peptides shown in Bold Red. The N-terminal 3xFlag tag and C-terminal last five amino acids of Flag-LC3B^G120A^ are underlined. The downward pointing arrow shows the LC3B start amino acid in the recombinant protein (first amino acid methionine was removed in the fusion protein). The arrow pointing upward shows the G120A point mutation in the Flag-LC3B^G120A^.

To determine whether Flag-LC3B^G120A^ is cleaved *in vivo*, we purified Flag-LC3B^G120A^ recombinant protein from HEK293 cells by Flag antibody-based affinity purification. Flag-LC3B^G120A^ was purified to a high degree as demonstrated by SDS-PAGE (Figure 4A, arrows). Elutions 2 and 3 were combined, concentrated and subsequently analyzed by top-down protein sequencing utilizing MALDI ISD mass spectrometry of intact proteins. MALDI ISD can confirm the amino acid sequence of 20–70 residues from both the N-terminal and the C-terminal ends of a purified protein [12]. The results of the Mascot software MS/MS ion search for the ISD spectrum of Flag-LC3B^G120A^ are shown in Fig. 4B–G. Sequencing of Flag-LC3B^G120A^ demonstrates that the faster migration band observed by Wb assay is indeed the full-length protein without cleavage.

### Human Pro-LC3B Migrates to a Similar Position as LC3B^G120A^ and LC3B-II by SDS-PAGE

**Figure 5:**
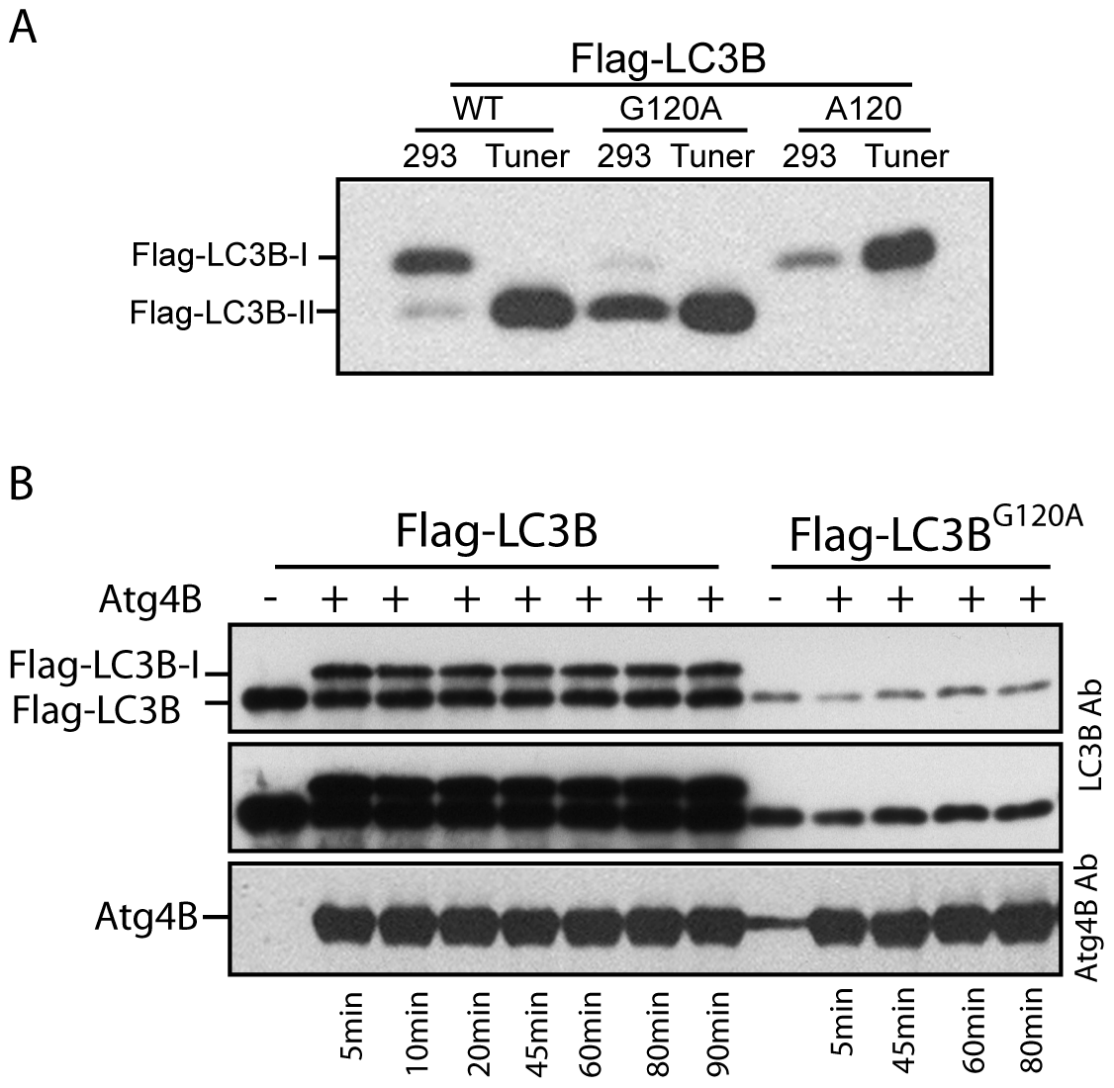
Pro-human LC3B migrates at similar rate as G120A mutant and lipidated form in SDS-polyacrylamide gel. ***A,*** pTriEX.1.1 plasmids encoding Flag-LC3B, Flag-LC3B^G120A^, or Flag-LC3B^A120^ was expressed in HEK293 or *E.coli* cells (Tuner cell), respectively. Recombinant protein expression in *E.coli* cells were induced with 1 mM of IPTG for four hours before harvesting. 10 µg of HEK293 or *E.coli* lysates were loaded onto SDS-polyacrylamide gel. Immunoblotting was conducted using Flag-antibody. ***B,*** 10 µg of *E.coli* lysates with Flag-LC3B or Flag-LC3B^G120A^ recombinant proteins was digested with recombinant Atg4B at 37°C for indicated time and analyzed by immunoblotting with LC3B antibody and Atg4B antibody as indicated.

The unexpected results thus far challenged our earlier hypothesis. To revise, we considered that should the LC3B^G120A^ band by Wb represent an unprocessed/uncleaved form of the protein, we expect that unprocessed human LC3B should have the same migration rate as LC3B^G120A^ as the only difference between these two forms of LC3B is the replacement of Glycine 120 by an alanine. As already demonstrated in this study, Flag-LC3B^G120^ migrates to the same position as Flag-LC3B^A120^ (Fig. 3A), in which Glycine 120 was replaced by an alanine. To validate our hypothesis, we sub-cloned Flag-LC3B, Flag-LC3B^G120A^, and Flag-LC3B^A120^ into pTriEX1.1 expression plasmid. This plasmid is equipped with promoters which can drive gene expression in both *E.coli* and mammalian cells. Flag-LC3B should not be cleaved when expressed in *E.coli* cell due to the absence of Atg4s in a bacterial cell. We expressed Flag-LC3B series recombinants in both HEK293 cell and *E.coli*. As shown in Figure 5A, Flag-LC3B demonstrates two bands in the HEK293 cells, which corresponds to Flag-LC3B-I and Flag-LC3B-II. Flag-LC3B expressed in *E.coli* migrated to be the same position as Flag-LC3B-II (Figure 5, WT/Tuner). Flag-LC3B^G120A^ migrated similarly as Flag-LC3B-II or unprocessed Flag-LC3B regardless of its expression in the HEK293 or *E.coli* (Fig. 5A, G120A). Flag-LC3B^A120^ expressed as same size bands in HK293 and *E.coli* cells as Flag-LC3B-I, verifying that Flag-LC3B^A120^ is not modified, which is consistent with the results already shown in this study.

As expected, these results reveal that human pro-LC3B is the same “size” as LC3B^G120A^. Of particular interest, the unprocessed human LC3B migrates at similar rate as LC3B-II by SDS-PAGE. Thus, the C-terminal five amino acids in human LC3B may affect the migration rate of LC3B rendering its faster movement than the smaller cleaved LC3B-I by SDS-PAGE.

To confirm the relative maintenance of function of the recombinant protein as expressed in a bacterial model, we incubated Flag-LC3B and Flag-LC3B^G120A^ bacterially expressed recombinants with recombinant Atg4B protein at 37°C for intervals as indicated in Figure 5B. Wb assay demonstrated the cleavage of Flag-LC3B by Atg4B as it gave rise to two distinct bands. The slower migrating band was actually the cleaved Flag-LC3B (Flag-LC3B-I) band and the faster migrating band was unprocessed Flag-LC3B. As a control, Flag-LC3B^G120A^ was not cleaved by Atg4B consistent with the data already shown in this study.

### The Last Five Amino Acids Determine Faster Migration of Human LC3B by SDS-PAGE

**Figure 6:**
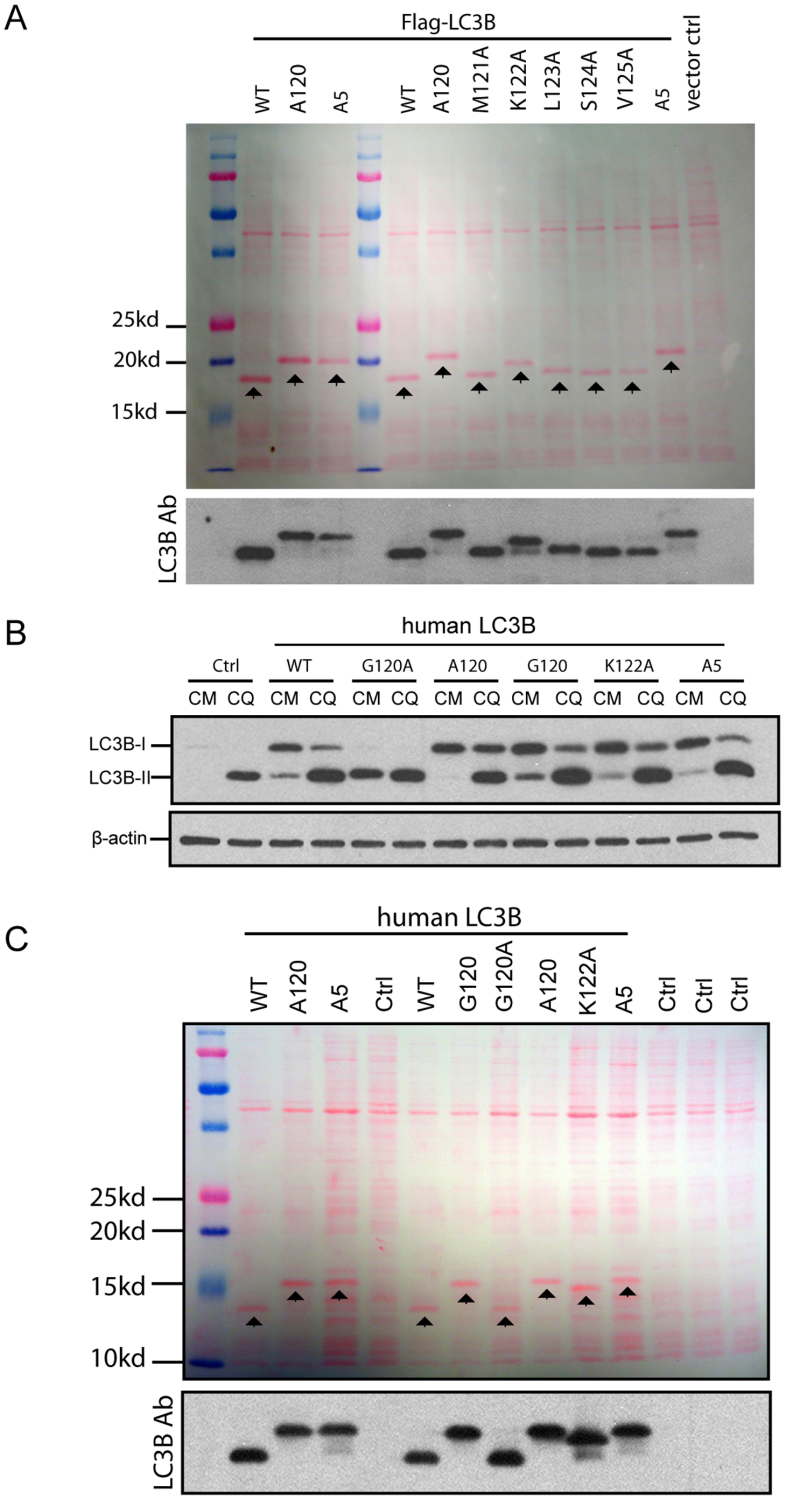
Last five amino acids determine human LC3B faster migration in SDS-PAGE. ***A,*** Flag-LC3B and indicated mutants were expressed in *E.coli.* 10 µg of cell lysates were then separated in 15% of SDS-polyacrylamide gel. Transferred membrane was stained with Ponceau S before immunoblotting with LC3B antibody. ***B,*** human LC3B and indicated mutants were expressed in HEK293 cells for 24 hours. Cells were then treated with 50 µM of CQ for 2 hours. Expression patters were analyzed by Wb using LC3B antibody. ***C,*** Same sets of expression plasmids were then expressed in *E.coli* cells. 10 µg of cell lysates were then separated in 15% of SDS-polyacrylamide gel. Transferred membrane was stained with Ponceau S before immunoblotting with LC3B antibody. Arrows point to wild-type and mutant LC3B recombinant protein band expressed in *E. coli.*

LC3B of human, mouse and rat origin have five amino acid residues after the Glycine at the 120 position. Investigation as to whether these five amino acids play a key role in pro-human LC3B migration behavior by SDS-PAGE was a subsequent experimental aim. Each individual amino acid after Glycine^120^ was replaced with Alanine to dissect the effect each amino acid residue had on Flag-LC3B migration. Expression of these mutant recombinant proteins in bacteria was analyzed by SDS-PAGE. Among the last five amino acids, Lysine^122^ and Leucine^123^ had the most significant effect on Flag-LC3B migration (Fig. 6A). K122A resulted in the slowest migration of Flag-LC3B followed by the L123A mutant and likely, therefore contribute the most to accelerated migration. M121A, S124A and V125A demonstrated less effect on Flag-LC3B migration. Exchange of all five amino acid residues to Alanine resulted in the slowest migration of Flag-LC3B (Fig. 6A, A5). This data supports the idea that all five amino acids likely contribute the faster migration behavior of Flag-LC3B. Of interest, the A5 mutant demonstrate a similar migration rate (Fig. 6A, A120) but not necessarily slower than Flag-LC3B-I. Such data suggests that the additional last five amino acids do not render unprocessed human LC3B to migrate slower than Flag-LC3B-I.

To exclude the possibility that the N-terminal tag influenced the previous findings, the K122A and A5 mutant were analyzed in the context of non-tagged human LC3B. Expression patterns of human LC3B, LC3B^G120A^ and LC3B^A120^ in HEK293 as previously illustrated in Figure 2C are used as band size references for LC3B^G120^, LC3B^K122A^ and LC3B^A5^ (Fig. 6B). LC3B^G120^, LC3B^K122A^ and LC3B^A5^ demonstrated similar band patterns and represent LC3B-I and LC3B-II as seen with wild-type LC3B (Fig. 6B, LC3B). All recombinant proteins showed an accumulation response following treatment with CQ. As such, these data demonstrate the amino acid residue exchange in K122A and A5 mutants do not affect its cleavage by Atg4B nor prevent normal passage through the lipdiation process.

These non-tagged LC3B were then expressed in *E.coli* cells to compare their size of unprocessed forms in SDS-PAGE gel. LC3B^G120A^ had the same size as wild-type LC3B (Fig. 6C, upper panel, G120A and WT). K122A mutant moved significant slower than pro-LC3B (wild-type) and A5 mutant migrated even more slowly with size similar to LC3B-I (Fig. 6C, upper panel, K122A and A5). Those LC3B expressed in *E.coli* cells were verified by immunoblotting with LC3B antibody (Fig. 6C, lower panel).

Altogether, these data demonstrate that the last five amino acids determine the accelerated migration rate of unprocessed human LC3B with Lysine 122 being a major contributing factor.

### Pro-mouse LC3B, -rat LC3B and -rat LC3 Migrate Faster than their Cleaved form by SDS-PAGE

**Figure 7:**
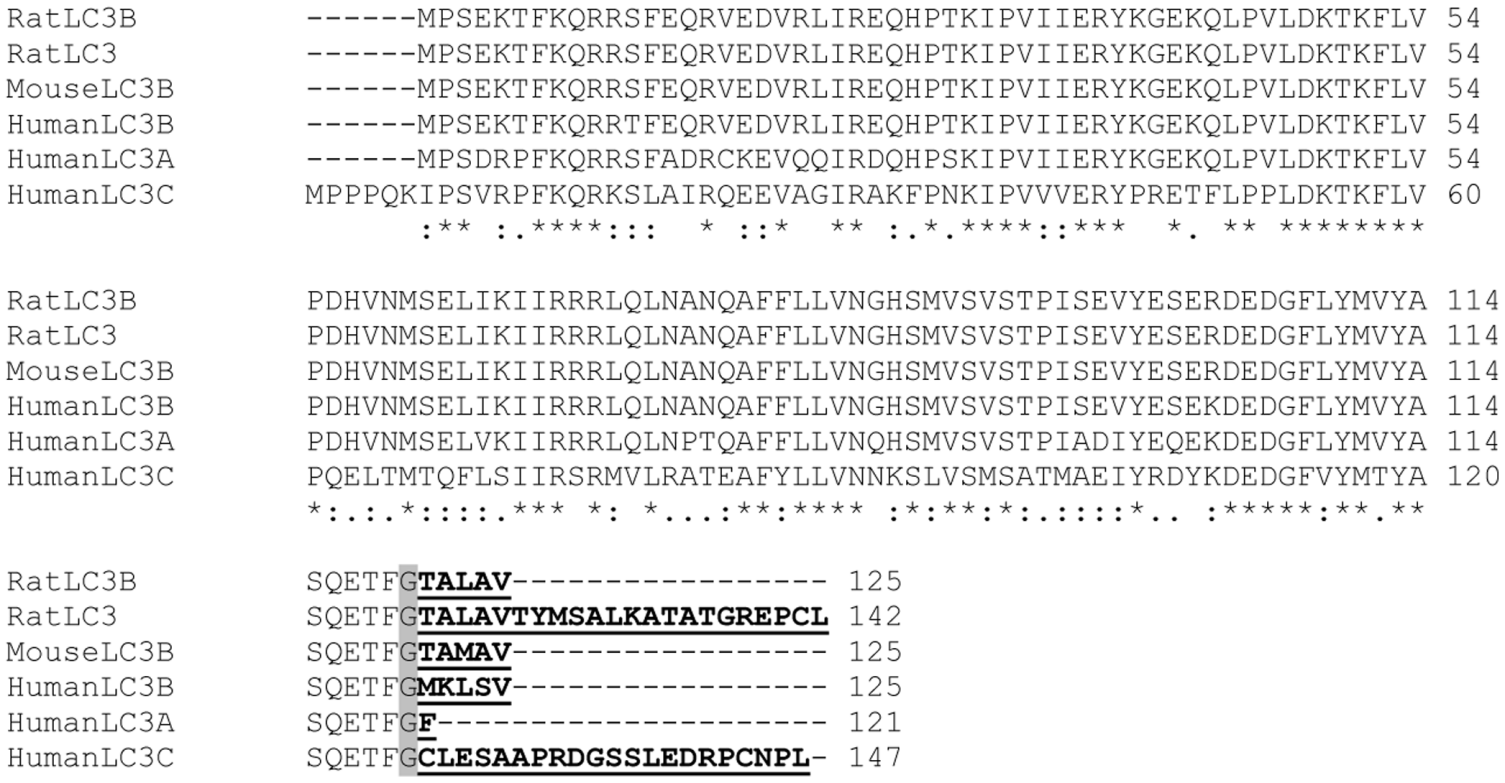
Sequence comparison of LC3 family member from human, mouse and rat origins. Pairwise alignments of LC3A, LC3B and LC3C of human and rodent origin are generated by ClustalW2. The glycine conjugation sites are boxed and C-terminal amino acids after glycine conjugation site are underlined. A “*” presents identity of amino acids; “**:**” presents these amino acids in a same amino acid group (similarity); “⋅” presents different group of amino acids.

**Figure 8:**
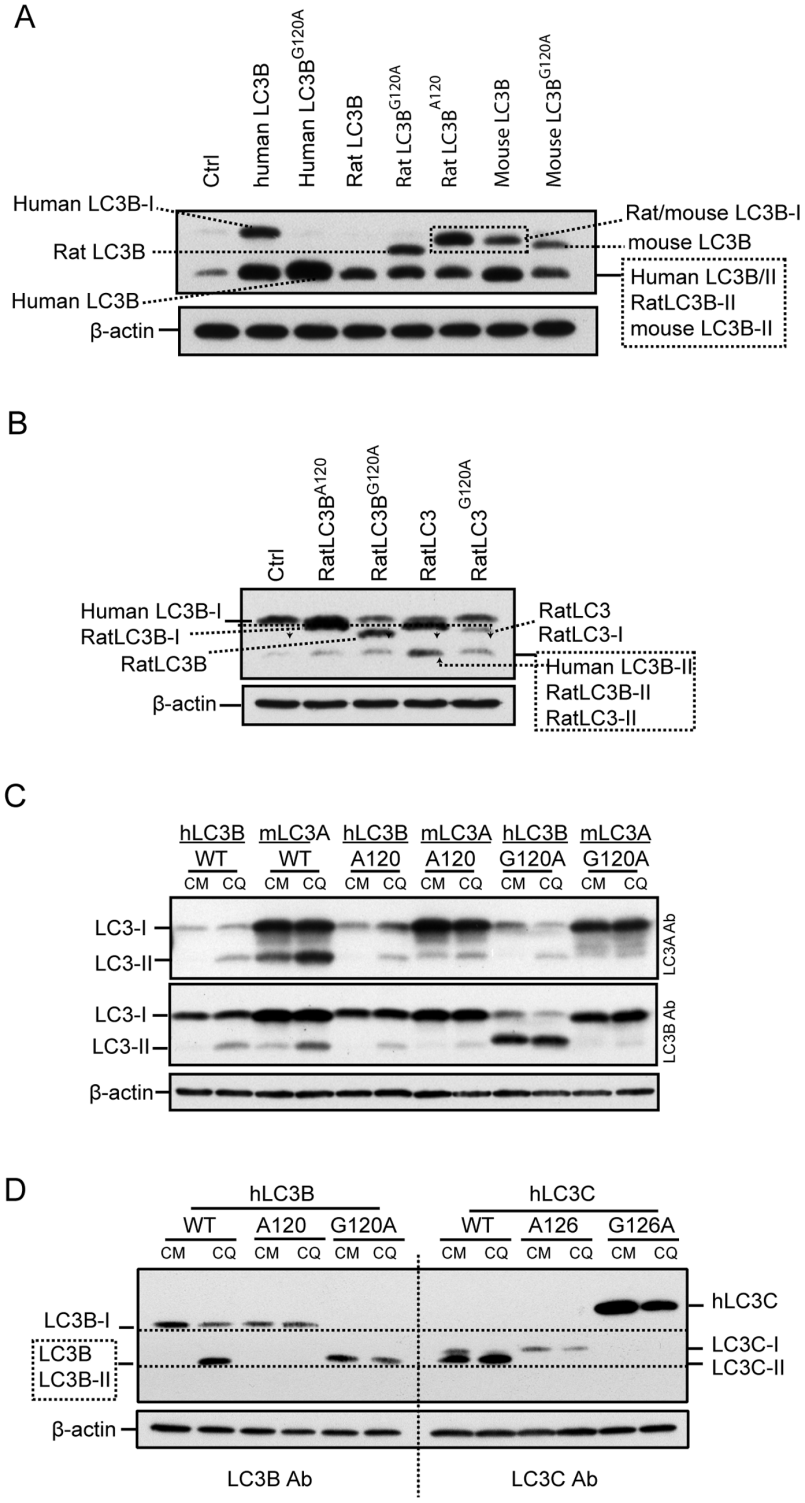
The faster migration is conserved in pro-LC3B of rodent species but not in human LC3C. ***A,*** Compare pro-human LC3B with pro-mouse LC3B, pro-rat LC3B and their cleaved and lipidated forms’ band patterns by 15% of SDS-polyacrylamide gel. Indicated expression plasmids were expressed in HEK293 cells. ***B,*** compare pro-rat LC3B with pro-rat LC3 and cleaved forms by 15% of SDS-polyacrylamide gel. Correspondent expression plasmids were expressed in HEK293 cells. Note: human LC3B-I band is close to RatLC3B-I/RatLC3-I as indicated by arrows. ***C,*** pro-LC3A and its cleaved form comparison by 15% of SDS-polyacrylamide gel. Indicated expression plasmids were expressed in HEK293 cells. ***D,*** compare pro-human LC3B and LC3C species by 15% of SDS-polyacrylamide gel. Indicated expression plasmids were expressed in HEK293 cells for 24 h. HEK293 cells transient expressing Human LC3C were treated with 50 µM of CQ for 2 h to induce accumulation of human LC3B-II and LC3C-II. Total cell lysates were separated by a 15% of SDS-polyacrylamide gel. Protein transferred membrane was cut between human LC3B group and human LC3C group and immunoblotting by LC3B and LC3C antibody, respectively. The immunoblotting membrane was re-aligned before developing with chemiluminescent substrate in order to compare human LC3B species and human LC3C species in Wb.

Has been demonstrated pro-human LC3B faster migration in SDS-PAGE, we further investigated whether the faster migration is conserved in murine and rat LC3B. We used G120A mutant to present uncleaved/pro-LC3B of rodent species in SDS-PAGE. Murine LC3B differs by only one amino acid from Rat LC3B as illustrated in Figure 7 (M123 versus L123), both share high amino acid sequence identity with human LC3B. Expression plasmids encoding rat LC3B, rat LC3B^A120^ and rat LC3B^G120A^ mutants, mouse LC3B and mouse LC3B^G120A^ mutant were constructed. As rat LC3B^A120^ and mouse LC3B^A120^ have the exact same amino acid sequence, rat LC3B^A120^ was used as a reference for both rat LC3B-I and mouse LC3B-I. Following expression of wild-type and mutant forms of human, mouse and rat LC3B in HEK293 cells, the cell lysates were separated by 15% of SDS-PAGE and probed using LC3B antibody. Human LC3B-I was found to migrate slower than both rat and mouse LC3B-I (Fig. 8A). Mouse LC3B-I and rat LC3B-I demonstrate equivocal band position by Wb which is consistent to their similar C-terminal amino acid sequence. RatLC3B^G120A^ represents an uncleaved rat LC3B in size and demonstrated slightly more rapid migration than that of mouse LC3B^G120A^ (equal to pro-mouse LC3B in size). Again, this data supports the previous finding in human LC3B that L123 as found in Rat pro-LC3B contributes accelerated migration.

Rat LC3 is an isoform of Rat LC3B with 22 amino acids long tail after G^120^ (Fig. 7). Rat LC3 has been widely used in GFP or RFP fusion expression vectors to monitor autophagy process [13]. As shown in Fig. 8 B, wild-type rat LC3 showed two bands in SDS-PAGE, slower migration band presents cleaved form rat LC3(rat LC3-I), which has equal size with rat LC3B^A120^ (equal to rat LC3B-I in size). Both rat LC3B^A120^ and rat LC3-I migrates slower than human LCB-I, consistent to the finding presented in the Figure 8A. Rat LC3B^G120A^ (equal to pro-Rat LC3B in size) migrated faster than rat LC3B^A120^ (equal to rat LC3B-I in size). Of note, rat LC3^G120A^ with 22 amino acids long tail and equal to pro-rat LC3 in size migrated faster than cleaved rat LC3-I/rat LC3B-I (Fig. 8.B).

Altogether, pro-mouse LC3B,-rat LC3B and –rat LC3 migrate faster than their cleaved forms. The faster migration is conversed in LC3B subfamily. However, compared to pro-human LC3B, pro-LC3B of rodent species migrate much slower than LC3B-II.

### Pro-LC3A has Same Migration Rate as LC3A-I by SDS-PAGE

Human, mouse and rat LC3A share same amino acid sequence. LC3A has a Phenylalanine (F) after G^120^ (Fig. 7). We compared murine LC3A (variant 1), LC3A^A120^ and LC3A^G120A^ (equal to pro-LC3A in size) recombinants with human LC3B expressed in HEK293 cells. As shown in Fig. 8.C, LC3A specific antibody detects only LC3A species (upper panels), while LC3B antibody recognizes LC3B and LC3A (middle panels). Transient expressed wild-type LC3A responded well to CQ treatment demonstrated by increase in LC3A-II band intensity. LC3A^A120^ (representing LC3A-I) and LC3A^G102A^ (representing pro-LC3A) showed band size equal to LC3A-I and LC3B-I. This result demonstrates that human, mouse and rat pro-LC3A migrates at same rate as LC3A-I; LC3A-I demonstrated same size to human LC3B-I by SDS-PAGE; and LC3A-II has no difference from LC3B-II in size by SDS-PAGE.

### Faster Migration Character is Not Conserved in Pro-human LC3C

Human LC3C shares less sequence similarity with LC3A (identity: 71/147; similarity: 90/147) and LC3B (identity: 67/147; similarity: 88/147). It has 21 amino acids long tail after glycine conjugation site (G126) (Fig. 7). No rodent version of LC3C was identified. To analyze whether the faster migration is preserved in pro-human LC3C, we transiently expressed wild-type human LC3C, LC3C^A126^ and LC3C^G126A^ mutants in HEK293 cells. We compared human LC3B species and LC3C species migration patterns in one 15% of SDS-polyacrylamide gel by immunoblotting with LC3B and LC3C specific antibodies. LC3C-I migrated dramatic faster than human LC3B-I and closed to LC3C-II (Fig. 8D). Of interest, LC3C^G126A^ mutant (Glycine 126 replaced by Alanine and equal in size to pro-LC3C) migrated much slower than LC3B-I and LC3C-I. This result demonstrates that LC3C species (pro-LC3C, LC3C-I and LC3C-II) have a unique migration patterns in SDS- polyacrylamide gel: LC3C-I migrates faster than all other LC3-I form in LC3 family and is close to LC3C-II. In contrast, pro-LC3C demonstrated the slowest migration specie in LC3 family. PE conjugation of LC3C does not render its significant faster migration than LC3C-I as found in other LC3 family members. It should be noted that LC3C-I and LC3C-II migrate too close to separate even by 15% of SDS- polyacrylamide gel. Thus, LC3C-I to LC3C-II conversion assay may not be an ideal marker for quantity assay in autophagy study. Of note, conjugation with PE renders LC3A-II, LC3B-II and LC3C-II a similar migration rate in SDS-PAGE independent on their primary amino acid sequences (Fig. 8A, B, C and D).

### Effect of Atg4B Expression Level on Pro-LC3B Cleavage *in vivo*

**Figure 9:**
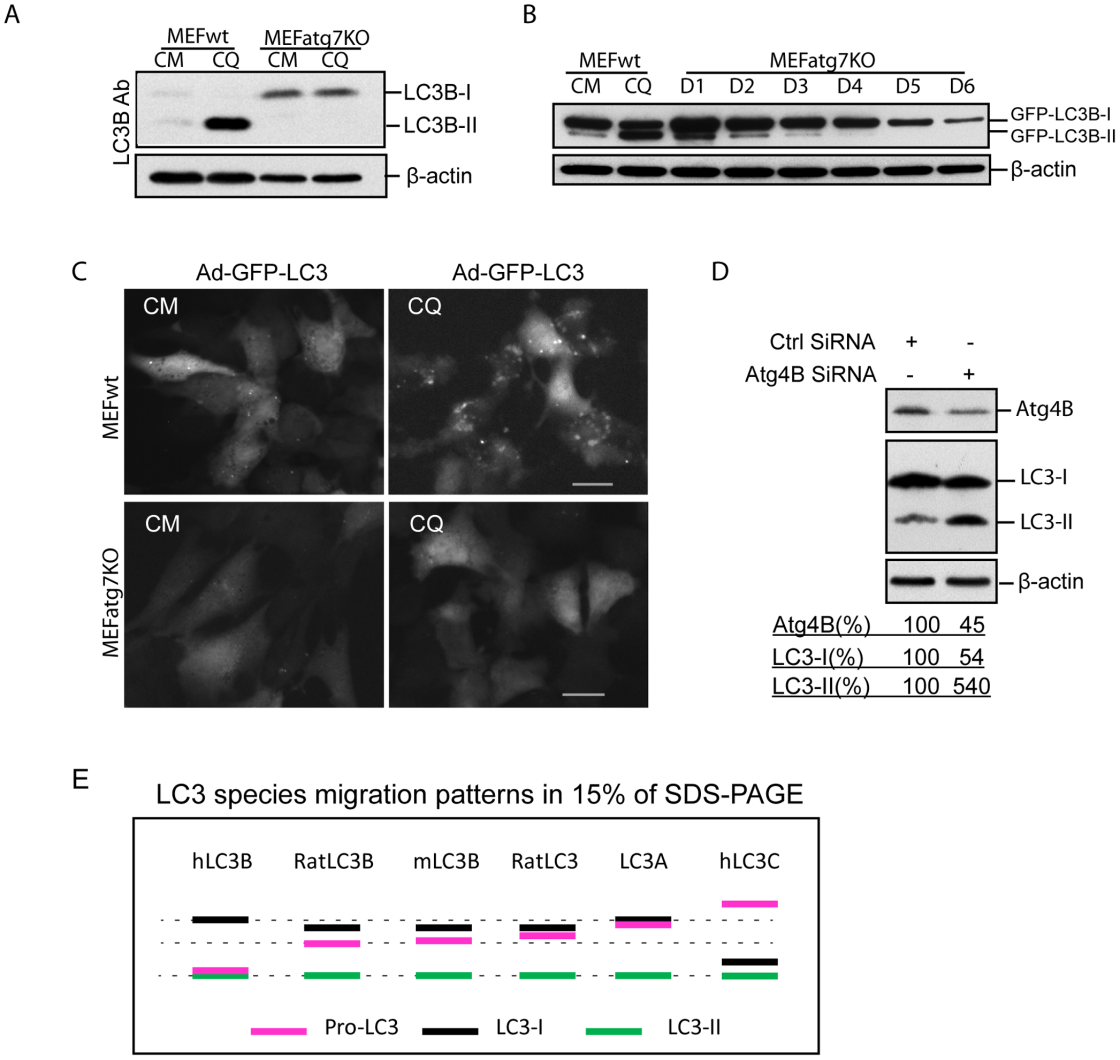
Effect of Atg4B on LC3B processing. ***A,*** MEFwt and MEFatg7KO cells were treated with 50 µM of CQ for 2 hours. LC3B expression patterns were then analyzed in 15% of SDS-PAGE by immunoblotting with LC3B antibody. ***B,*** Adenoviral vector expressing GFP-LC3B was used to infect MEFatg7KO cells in series double diluted doses for 24 hours (D1 = 20MOI). Ad-GFP-LC3B (20MOI) infected wild-type MEF cells were used as control or treated with 50 µM of CQ for 2 hours.GFP-LC3B expression patterns were analyzed by 15% of SDS-PAGE. ***C,*** Ad-GFP-LC3B infected MEFwt and MEFatg7KO cells were treated with 50 µM of CQ for 2 hours and images were recorded by fluorescence microscopy. ***D,*** HEK293 cells were transfected with Atg4B siRNA and Control siRNA for 48 h. 15 µg of total cell lysates were analyzed by immunoblotting with Atg4B, LC3B, and β-actin antibodies. Image J software was used to quantify band density using β-actin as loading control. ***E,*** Diagram of LC3 species migration patterns in 15% of SDS-polyacrylamide gel. The diagram summarizes LC3 species (pro-LC3, LC3-I and LC3-II) band patterns from human, mouse and rat origins. Refer to the result section for description in details.

Pro-LC3B is efficiently cleaved by Atg4B following translation. To examine the possibility that Atg4B may not fully process over-expressed LC3B, we utilized an Atg7 knockout MEF cell model. Without the ability to form LC3-Atg7 intermediates within MEFatg7KO cells, the LC3B lipidation process is terminated at the LC3B-I stage. As expected, no LC3B-II was detected in MEFatg7KO cell when compared to wild-type MEF cell (Figure 9A). Unprocessed/uncleaved GFP-LC3B was clearly visible at the expected GFP-LC3B-II band location in MEFatg7KO cells following transduction with an adenoviral human GFP-LC3B vector (Figure 9B; D1 to D4). Despite the increase in unprocessed GFP-LC3B in MEFatg7KO cells there was no discernable GFP-LC3B punctation when compared to GFP-LC3B punctuation in wild-type MEF cells (Figure 9C), demonstrating unprocessed GFP-LC3B migrates similar as GFP-LC3B-II and distributes in the cytosol.

Reversely, low Atg4B level may negatively affect pro-LC3processing. HEK293 cells were transfected with Atg4B siRNA to knockdown Atg4B. As shown in Fig. 9D, Atg4B was reduced by 55% at 48 hours post transfection which resulted in 45% decrease in LC3-I and 540% increase in “LC3-II”. These increased “LC3-II” are from unprocessed LC3B.

Taken as a whole, these data demonstrate that unprocessed LC3B can be detected in certain conditions such as over-expression of LC3B and lower level expression of Atg4B. These findings are particularly important for Atg4 inhibitor assays in which blocking Atg4 activity should lead to accumulation of pro-LC3B.

## Discussion

In this study, we demonstrated that unprocessed human LC3B migrates at a similar rate as LC3B-II in SDS- polyacrylamide gel. The faster migration behaviors were conserved in pro-mouse and pro-rat LC3B although they do not shift to LC3B-II location as found in human LC3B. Dramatically, even with 22 amino acids long C-terminus after Glycine 120 (about 18.3% longer than rat LC3-I, Fig. 7), pro-rat LC3 migrates faster than rat LC3-I. These findings experimentally clarify the pro-LC3B band location in SDS-PAGE.

In general, protein size is linearly proportional to its number of amino acids once protein binds SDS and run in SDS-polyacrylamide gel [14]. There are exceptions of this rule such as membrane bound protein with alpha helix structures shows gel shift in Wb assay [15]. However, no secondary structure was found in the C-terminus after G^120^ of LC3B proteins. Thus, it is logical to presume that pro-LC3B should migrate slower than LC3B-I because of five more amino acids at C-terminus. However, this was proven wrong by detailed analysis of human LC3B in molecular level by SDS-PAGE. Our findings, for the first time, demonstrate that the last five amino acids in human LC3B, particularly Lysine and Leucine residues, change the pro-LC3B migration behavior leading to its faster migration than LC3B-I in SDS-PAGE. The significance of the last five amino acids renders human pro-LC3B indistinguishable from LC3B-II in Wb.

Four human LC3 genes (LC3A, LC3B, LC3B2 and LC3C) have been identified. No LC3B2 and LC3C were found in rodent species. LC3B2 has only one amino acid different from LC3B (Y^113^ versus C^113^), and has very limited tissue distribution [16]. LC3A has two variants. LC3A variant 1 seems to be widely expressed among tissues compared to variant 2 [7], [16]. Interestingly, LC3A variant 1 was found frequently inactivated in human cancers by aberrant DNA methylation and associated with carcinogenesis. LC3C is transcribed at lower levels with limited tissue distribution [7], [16]. LC3C was found to selectively bind NDP52 and was required for antibacterial autophagy [17]. There is no detailed quantity information on LC3A, B and C expression in a particular type of cells. The ratio among LC3A, B and C, if they are co-expressed in a cell, is unclear. Of note, we find that LC3B is well response to chloroquine treatment in several cancer cell lines (A549, HCT116 and TPC-1) where LC3A and LC3C are lack of significant increase in LC3A-II or LC3C-II level (unpublished data), indicating LC3 family members may differentially involve in autophagy-related processes.

As we demonstrated in this study, Atg4B level can affect pro-LC3B level. Cells from variant tissue origins may have different levels of Atg4B expression and Atg4B activity may be affected by other factors [18]–[22]. Unprocessed human LC3B level could affect the LC3B-II analysis in certain human cells and tissues in certain conditions. Under such conditions, the LC3-II level may not truly reflect LC3B-II specie, i.e., the LC3-II observed by Wb assay could be partly from the unprocessed LC3B. In this situation, subcellular fractionation is recommended for human LC3B-II assays.

A number of research groups are working on searching for Atg4 inhibitors. Assays for evaluation of Atg4 activities *in vivo* and *in vitro* were developed based on C-terminal tags of LC3B including LC3B-GFP [23], LC3B-phosphlipaseA [24], FRET based CFP and YFP tagged LC3B [25], LC3B-luciferase [26]. These assays were developed for informative indicator for autophagy process or for searching for Atg4 inhibitors. It is interesting to note that none of these Atg4 inhibition assays are based on analyzing pro-LC3 level. One reason is likely due to unknown band patters for pro- LC3 in Wb. It would be particularly confused by looking at pro-human LC3B band in these studies, where pro-LC3B is localized at the LC3B-II site in Wb. Our finding could apply for these situations, for instance, a recent report on Atg4B knockout mouse’s LC3 Wb assay where close two bands apparently representing murine LC3-I in tissues from Atg4B knockout mice may actually represent mouse LC3B-I and unprocessed LC3B, respectively [27].

In conclusion, we find that the C-terminal amino acids after glycine conjugation sites determine pro-LC3B’s migration behaviors in SDS-PAGE. Figure 8E summarizes LC3A, LC3B and LC3C species band patters in 15% of SDS-PAGE for human, mouse and rat origins. For human LC3B, pro-LC3B is indistinguishable from LC3B-II; pro-human LC3A migrates to the same site as LC3A-I; pro-rat LC3B migrates faster >pro-mouse LC3B>pro-rat LC3> rat LC3-I/rat LC3B-I/mouse LC3B-I; mouse and rat LC3B-I migrate a little faster than human LC3-I; Human LC3C-I migrates faster>human LC3B-I and human LC3A-I >pro-human LC3C; LC3A-II and LC3B-II migrate at similar rate as LC3C-II. Overall, the data presented provide a guide for cell-based or animal model Atg4 knock out/knock down and Atg4 inhibitor assays as it relates to the measurement of pro-LC3, LC3-I and LC3-II by immunoblot analysis.
